# Open Book Exams and Flexible Grading Systems: Post-COVID University Policies from a Student Perspective

**DOI:** 10.3390/bs13070607

**Published:** 2023-07-21

**Authors:** Dongsuk Kang

**Affiliations:** Department of Business Administration, College of Social Sciences, Gangneung-Wonju National University (GWNU), Street 7, Jukheon-gil, Gangneung-si 25457, Republic of Korea; professional@gwnu.ac.kr

**Keywords:** course management, educational innovation, university management, education service, higher education

## Abstract

Due to COVID-19, many universities have started offering real time video or recorded courses. This situation raises concerns about a decline in students’ learning outcomes and issues of unfairness regarding students’ exams and grade evaluations. Korean universities have introduced online open book testing in courses and a flexible pass grading system that allows students to select their final grades to improve fairness. This research investigates students’ thoughts and reasons for the test and the system through a questionnaire with 109 respondents and statistical methods such as nonparametric tests, multinomial regression and text-mining. Many students supported both the testing and the grading system, presenting balanced viewpoints by comparing their cons and pros. This finding suggests that these policies could be helpful in enhancing fairness in grade evaluation, relieving students of the learning burden and increasing their satisfaction. This study offers the implications that universities must develop standardized exam formats as well as various learning options in a rapidly changing situation with educational innovation (e.g., hyperscale and generative artificial intelligence).

## 1. Introduction

In response to the spread of the COVID-19 virus, many universities worldwide have adopted remote courses as a necessary measure to cope with social distancing and containment of infectious diseases. This shift has led to significant controversy surrounding the measurement and evaluation of learning performance and grading outcomes in remote courses. For example, there has been an increase in cheating behaviors whereby students are required to do an assignment or exam individually but instead solve quizzes together and share answers through instant messaging or offline meetings [1,2] in South Korea or the Republic of Korea (Hereinafter referred to as South Korea). This problematic situation has raised concerns about the unfairness of learning performance assessments and the distrust in grade evaluation about remote learning, creating a vicious cycle of public distrust in remote courses and their grades [1] as well as student dissatisfaction with university life and potential dropouts. Moreover, the rapid shift to online courses due to the global spread of COVID-19 and social distancing policies has led to negative perceptions among students and instructors regarding a general decline in course quality [3].

In this complex environment, enhancing the fairness of learning evaluations in remote courses has become a critical issue for higher education institutions, which are faced with societal expectations for improved course services. Many universities have implemented open book exams in a real time and online environment and/or flexible pass grading systems. Some Korean universities have been using a flexible pass grading system since the spring semester of 2020. Students can select their course outcomes and whether they obtain their original grades or “pass” if their grades are higher than D0 [4]. This system has the merits of alleviating the controversy over the fairness of students’ grade evaluation due to cheating in the weak proctor environment [4].

It can also have the demerits of inflating students’ grade point averages and decreasing their motivation, which can prompt them to abandon difficult courses and not study hard for exams. These two alternatives (i.e., open book exams and a flexible pass grading system) would be temporary and adaptive policies of course management in response to students’ movement toward tuition refunds as they have repetitively argued about their disappointment in the quality of courses [5,6,7,8]. This problematic situation highlights the need for relevant research about the innovation of education services and policies concerning learning assessments and grading systems in higher education. However, the literature has primarily focused on specific topics such as the impact of open book exams and/or the online proctor on students.

Therefore, this study proposes three relevant research questions addressing students’ perceptions and their reasons for open book exams in real time and online environments as well as flexible pass grading systems. What opinions and reasons do students have regarding open book exams? (Research Question 1, RQ 1) What are the perspectives of students who have experienced real time and online open book exams? (RQ 2) What opinions do students have about the flexible pass grading system and what are their reasons? (RQ 3)

A questionnaire on the opinions of open book exams and flexible pass grading systems and their reasons was conducted among 109 students who took an online and real time open book exam. Statistical and content analyses revealed that more than half of the students expressed positive opinions on the exam and the system. The primary reasons for accepting the exam included the easy use of course content, the reduction of their learning burden and better understanding of the content learned. Students’ main reasons for supporting the grading system were the increased possibility of minimizing online cheating, the decrease in their learning burden due to remote courses and the plausible enhancement of fairness in grade evaluation.

These findings suggest that instructors and universities need to improve their course and evaluation systems to enhance students’ satisfaction with their courses and university life. Student dissatisfaction can lead to increased dropout rates in the challenging environment of remote courses and the ongoing COVID-19 crisis. This study offers meaningful insights to treat and analyze students’ judgments and detailed reasons concerning real time and online open book exams and flexible grading systems.

## 2. Literature Review

### 2.1. The Adoption of Online Education and Open Book Exams

Online education and open book exam have been adopted as some tools for educational innovation. Many universities have implemented electronic learning (e-learning) via the internet, distance learning provided by open universities and massive open online courses (MOOC) in the 2000s [9]. Some motivations for this online transformation would be the expansion of students (e.g., office workers, freelancers) and the reduction in labor costs caused by offline education [9]. On the other side, an open book exam is a form of process-oriented learning and evaluation; it can promote learners and instructors to participate in exams by making more creative or realistic problems and solving them with combinations of knowledge they have learned [10].

Many countries have implemented online learning as an educational response to the lockdown and social distancing caused as a result of COVID-19 [11]. This transformation to online education can have several advantages: students’ learning safely in their accommodation, the reduction in the burden to prepare offline textbooks and materials and plausible high interaction between instructors and learners via telepresence technologies [12].

This educational change can bring some difficulties: possible weak feedback between instructors and learners due to their inexperience in using the technologies, students’ feeling of loneliness and/or low level of self-control in their learning and students’ different environment in terms of internet access and devices [11,13]. Moreover, some developing countries (e.g., India) have been confronted with regional digital divides among students in terms of discrepancies in social diffusion of wired/wireless internet and available electronic devices for online learning [12].

### 2.2. The Development of Online Education and Exams after COVID-19

After the global outbreak of COVID-19, universities adopted diverse formats or models of online education due to the development of educational technologies (e.g., EduTech or EdTech). For example, an instructor can choose a single or mixed format for online learning such as a traditional approach of uploading recorded courses to an e-learning management system, real time courses with telepresence services (e.g., Webex, Zoom) or blended learning that utilizes a recorded video, real time classes and instant messenger services (e.g., WhatsApp, KakaoTalk) for educational purposes and close student feedback [11,14]. These various forms of online education can mitigate some constraints of space and time associated with traditional courses [14,15,16]. This technology-driven educational innovation can promote diverse course activities such as traditional assignments, team-based projects and online exams/quizzes.

Exams under online learning due to COVID-19 have changed from offline paper-based tests to an online format of evaluation. For example, universities have adopted electronic exams (i.e., eExams), in which learners complete tests via their personal computers or electronic devices using a standardized evaluation system [15]. This method can reduce administrative costs for test execution and evaluation [15].

Open book exams have also been implemented in the online format (e.g., quizzes and final exams through e-learning). In particular, this exam format has been actively implemented in medical and other relevant universities that require students to study rigorously and take complex tests [17,18]. Exams can encourage students to learn and practice knowledge by reducing the burden of memory and focusing on organizing and utilizing knowledge [18,19]. Students can engage in deeper learning by solving problems similar to real-life situations, where doctors and nurses treat a patient without knowing the exact cause of their illness, requiring them to find and integrate information and knowledge regarding their illness/disease and treatment [18,19].

### 2.3. Critical Issues in Online Open Book Exams: Academic Integrity and Grading System

When implementing online open book exams, ensuring academic integrity could be critical for students to achieve their outcomes from online learning. Students may be unfamiliar with these exams and they may be tempted to cheat or engage in academic misconduct due to the instructors’ difficulty in online proctoring [16]. For example, Korean universities have discovered several significant cases of cheating in medical and engineering colleges, where two or more students shared their problem sets and answers through instant messaging during remote midterm exams in 2020 [1,2]. At some points, students can use course materials or summaries to understand and solve questions during the exam, which may prompt them to study less and/or be overconfident about the exam [19].

Such exams can also require technological and policy preparation for academic integrity. For example, various methods of recording and verifying students’ biological authentication (e.g., fingerprint, eye movement) and information on internet access (e.g., IP address) could be necessary on multiple occasions before and during the examination as anticheating measures [20]. Other technological controls of students’ behavior (e.g., rehearsal of pilot tests for final exams, antibacktracking of problem-solving, problem reshuffling) and error-correction (e.g., solutions for freezing/glitching during the exam) could be plausible results of close cooperation between instructors and ICT supporting teams [21]. Furthermore, strong policies and punishments against cheating and academic dishonesty could be imperative because this official notice could warn students to minimize their incentives and opportunities to engage in such undesirable behavior before and during the exam [20].

The grading system could be important for instructors and students as one of the post-evaluation policies. The exam requires both instructors and students to invest much time in formulating and solving creative problems, respectively, which could result in undesirable outcomes of difficult exams and poor grading due to both educators’ and pupils’ unpreparedness [12]. In addition, if an instructor does not have sufficient experience in online education or does not have adequate feedback for students, these studentsa’ learning performance would be lower than that of students in offline courses pre-COVID-19 [13].

As a practical policy addressing quality issues in online learning, some Korean universities have implemented a flexible pass grading system in which students can choose their course grade between their original scores or pass if the scores are higher than D0 [4,5,6]. However, these educational policies at universities could be fundamentally ineffective for controlling cheating and may be closer to a temporary and rewarding response to managing student complaints.

## 3. Materials and Methods

### 3.1. Study Design

**Figure 1:**
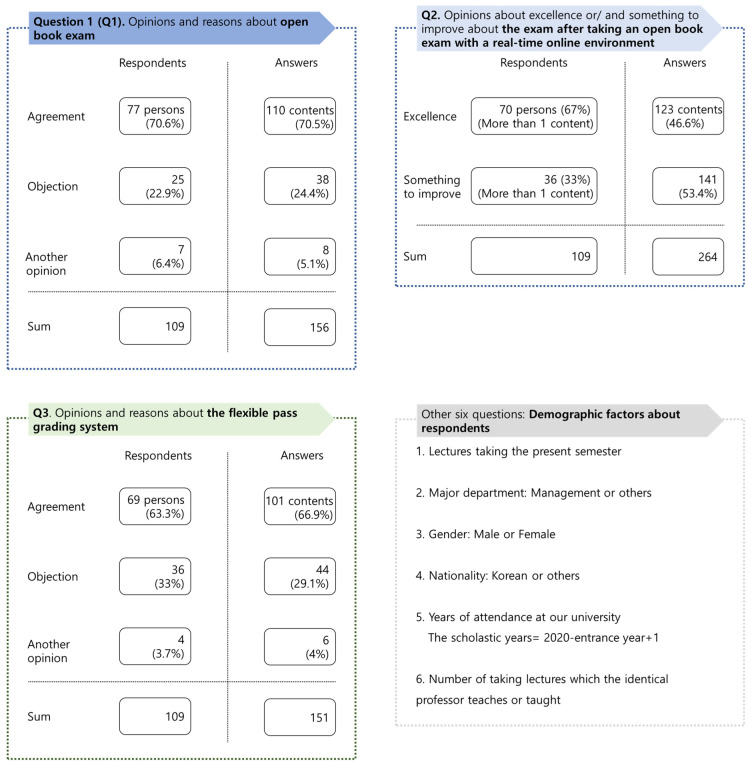
Survey structure of this research.

Drawing on a review of the literature concerning open book exams and grading systems after the outbreak of COVID-19, this study designed a survey to explore students’ opinions related to the open book exam and the flexible pass grading system and their reasons (Figure 1). Sample data were obtained after students completed their final exams in the form of open book exams within a real time and online environment.

As the instrumentation and the design for survey and data collection, this study employed a two-part questionnaire addressing the exam and the grading system after reviewing the relevant literature: (1) open-ended questions (e.g., opinions and reasons) about the exam and the grading system, as well as a review of the exam after taking it online and (2) closed-end questions about students’ demographic information. In the first part, students gave their opinions regarding each topic and their reasons in the open-ended questions, giving their reviews about excellence and/or ways to improve for the final exam performed in a real time and online format. In the second part, students provided their demographic characteristics such as their first major, gender, year of enrollment and the number of courses attended in the business department. Approval of the survey from the research committee was not necessary for this study because it falls within the scope of exemptions supported by the Bioethics and Safety Act (Article 13) in South Korea [22]. This category includes research projects that do not involve sensitive information and where research subjects cannot be personally identified [22].

**Figure 2:**
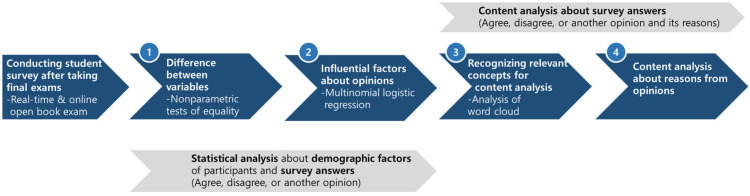
Methodological flow of the research: statistical tests, analysis of word cloud and content analysis.

This research utilizes four methods of statistical equality tests, multinomial logistic regression, word cloud analysis and content analysis to investigate students’ opinions of open book exams and their reasons, reviews after taking a real time and online open book exam and the flexible pass grading system (Figure 2). This research explains the methods in the Section 3.7.

### 3.2. Setting

This research settled the questionnaire (Figure 1) based on the literature review. The study conducted the questionnaire in Korean using Google. The research gathered its sample during the final exam periods (from 30 June to 2 July 2020, from 15 to 18 December 2020) to obtain a sufficient sample size. These were students who attended business administration courses at a national university in Gangwon State, South Korea.

### 3.3. Participants

Before beginning the survey, this research announced to students that the survey was conducted with informed consent and their information was only used for the present research purposes according to Korea’s Personal Information Protection Act [23]. The study surveyed 120 students who took real time and online open book final exams for six different classes.

### 3.4. Variables

After reviewing the relevant literature, this research identified two groups of demographic factors to influence students’ perception of the exam and the system. The first group consists of two numerical variables: students’ scholastic years and the number of courses they are taking. The second group incorporates four categorical variables: their major, gender, nationality and course semester. This research explains the descriptive statistics of the variables in the Section 3.6.

### 3.5. Bias Control

**Figure 3:**
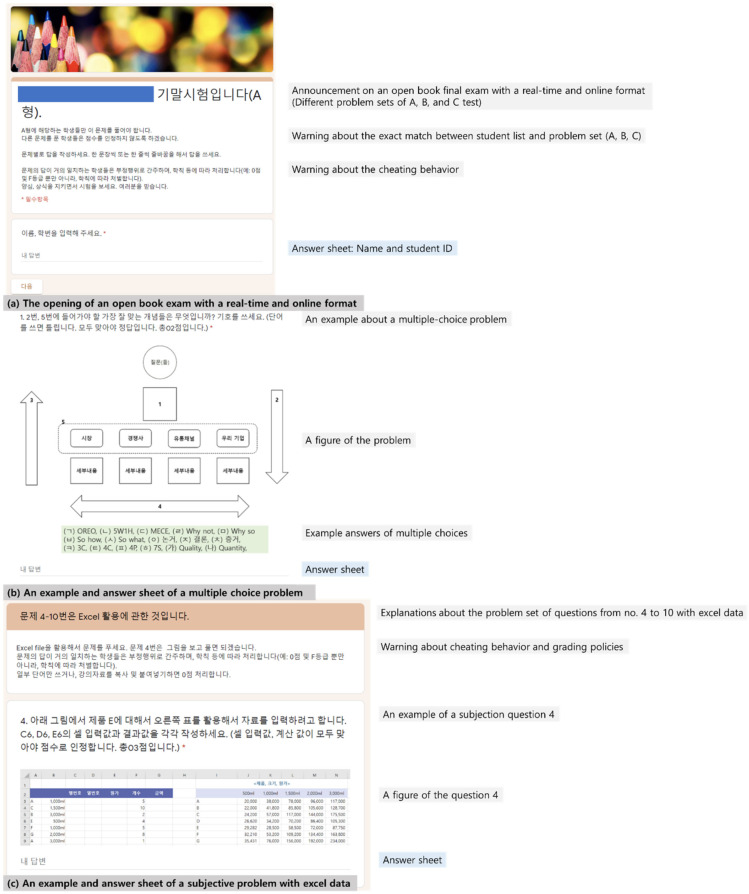
An example of an open-book final exam with a real time and online format of Google survey.

Before conducting a survey on students, this study performed a real time and online open book exam (Figure 3) through Google in the same way as the survey and utilized various treatments to ensure fair conditions for the test. Above all, for each course, the research established different sets of problems (e.g., A-B formats) according to the number of students. The study requested students to use their webcam and announced anticheating and grading policies several times before the test and on the day of the test. The research disclosed the problem sets and the relevant list of students by problem set during the exam.

### 3.6. Sampling and Study Size

After completing the survey (120 pupils), the survey data of 11 students who did not completely answer the questionnaire were removed. The final response rate was 90.83% (=109/120 students). Descriptive statistics show that 109 students participated in the questionnaire and the majority (70.6%) expressed approval for the open book exam. Many students offered positive reviews (i.e., excellence at 67%) for the exam with a real time and online format. More than 63% of students favored the flexible pass grading system; in addition, looking at the demographic variables of the respondents (Table 1: Panel A and B), many students majored in business administration (81.7%). Some differences were detected among the respondents with regard to the six classes, and most respondents (98.2%) were Korean. The respondents for the first and second semesters were also relatively equal. Although this research does not show the correlation table of all variables due to length limitations, the linear relationship between variables seems to be relatively low. This research selected the variable of “semester” as a control variable for multinomial logistic regression as the number of datasets in it is larger than that of the factor of “course number”.

### 3.7. Statistical Methods

As mentioned in the Section 3.1. (Figure 2), this research adopts four methodologies to analyze and understand students’ opinions of the exam and the grading system and their reasons. As the first method, a statistical test was performed to detect any difference in the demographic characteristics of students according to their opinions on the open book exam and flexible pass grading system. The research implemented several nonparametric statistical tests of equality of at least two independent groups in the case of non-normal distribution of data values [24,25,26,27].

As the second method, this study executed a multinomial logistic regression analysis to determine how students’ demographic variables affect their opinions of the open book exam and flexible pass grading system, respectively. According to Poursheikhali Asgary, Jahandideh [28], the current study adopted Equations (1) and (2) to perform the logistic regression in questions 1 and 3 (Figure 2). The dependent variable is students’ opinions about the respective question; if they expressed their agreement to the question, the probability of the agreement is $\mathrm{Pr}\left(j=1\right)$ in Equation (2). If they expressed their disagreement t the question, the probability of the disagreement is $\mathrm{Pr}\left(j=2\right)$ in the same equation. The research defines the probability of students’ opinions about another opinion as the probability of the reference group or option. The independent variables are students’ demographic factors (Table 1). This study utilized STATA (IC. Version 14.2) and R software (e.g., KoNLP) for statistical methodologies and the analysis of the word cloud.

(1) $$ln\left(\frac{Pr\left(j\right)}{Pr\left(0\right)}\right)=\beta_{0}^{}\left(j\right)+\overset{}{\underset{}{\sum }}\beta_{i}^{}\left(j\right)X_{i}^{}.$$

* Notes. $\beta_{0}^{}\left(j\right)$: an intercept at *j* = 1, 2. $\beta_{i}^{}\left(j\right)$: an independent variable *i* at *j* = 1, 2.

(2) $$Pr\left(j\right)=\frac{exp\left(\beta_{0}^{}\left(j\right)+\sum_{}^{}\beta_{i}^{}\left(j\right)X_{i}^{}\right)}{1+\sum_{j=1}^{2}exp\left(\beta_{0}^{}\left(j\right)+\sum_{}^{}\beta_{i}^{}\left(j\right)X_{i}^{}\right)}$$

* Notes. *Pr*(0): probability of group 0 (or a reference group) that students expressed another option about a question.

*Pr*(1): probability of group 1 that students expressed agreement about a question.
*Pr*(2): probability of group 2 that students expressed disagreement about a question.

As the third method, keywords from the reasons behind the students’ respective opinions were analyzed. This study utilized R packages related to Korean language such as KoNLP [29,30,31]. The study set was the dataset written in Korean with words repeated at least twice in each opinion.

As the last method, this research categorized and implemented content analysis in detail using the keywords of the word cloud analysis in each topic of the open book exam, real time and online open book exam and flexible pass grading system. This method could complement the word cloud analysis of a quantitative method, which could explain students’ reasons or concrete thoughts about their opinions of open book exams and flexible grading systems.

## 4. Results

This study analyzed students’ opinions on the open book exam and their reasons, reviews about the exam and the flexible pass grading system through four relevant methods (Figure 1). Firstly, this study implemented a statistical test about differences in the demographic characteristics of students based on their opinions on the open book exam and flexible pass grading system. Because opinion variables do not follow a normal distribution (Figure 2 and Table 2: Panel A), the research implemented several nonparametric statistical tests. As a result of the analysis, the difference between students expressing agreement and disagreement was not significant in general, except for a few variables (Table 2: Panel B).

Secondly, this study performed a multinomial logistic regression analysis about how students’ demographic factors can influence the opinions of the open book exam and flexible pass grading system. The study implemented the analysis with the options of robust regression and a significance level of 0.05 (Table 3). As a result of the analysis, when comparing students favoring the open book exam with those holding another opinion, the dummy variables of the foreign students and the second semester had a positive impact on the students who agreed with the exam.

On the contrary, compared to the students holding another opinion, the dummy variable of the business major has a negative impact on the students who disagreed with the exam. In the case of the flexible pass grading system, the dummy variable of foreign students had a statistically positive influence on the students who agreed with the system; however, in the case of the students who objected, there were no statistically significant variables.

**Figure 4:**
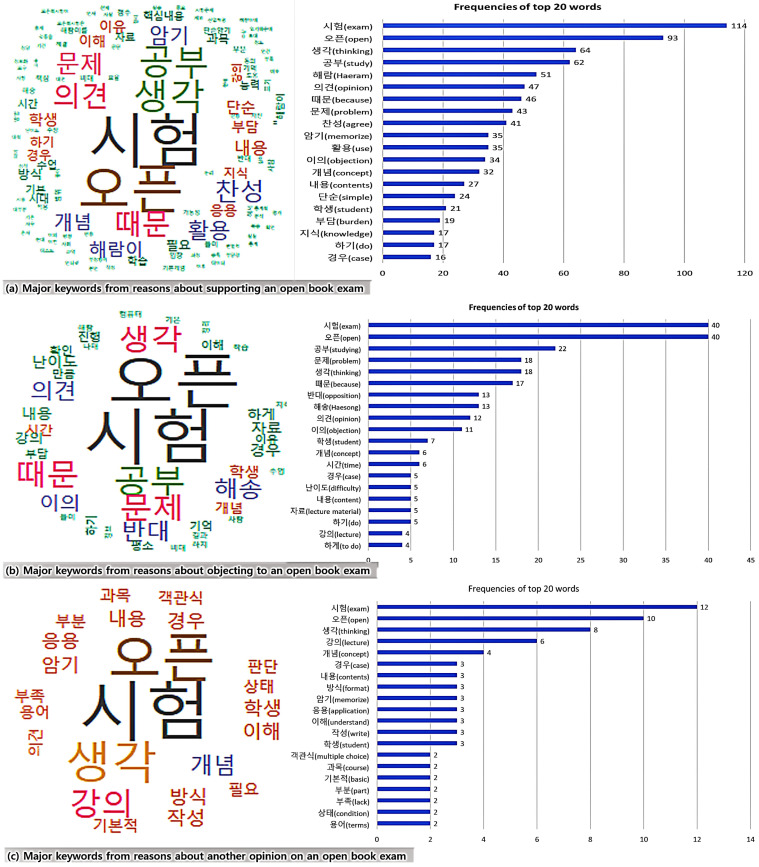
Analysis of word cloud: top 20 keywords of opinions and reasons about open book exams.

**Figure 5:**
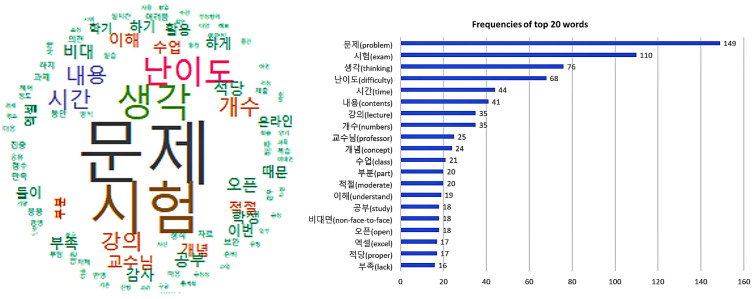
Analysis of word cloud: top 20 keywords of opinions about the exam after taking an open book exam with a real time and online environment.

**Figure 6:**
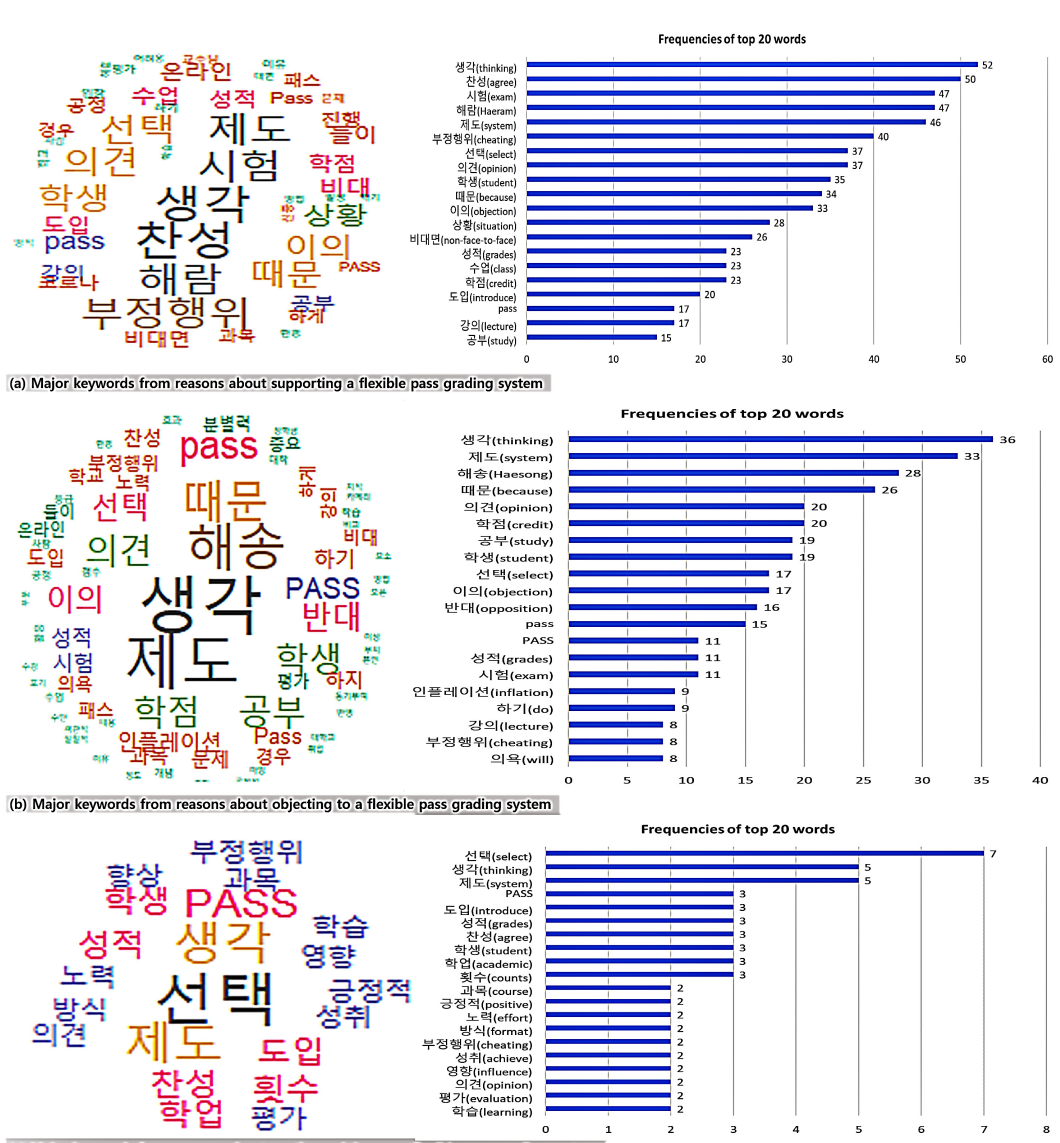
Analysis of word cloud: top 20 keywords of opinions and reasons about a flexible pass grading system.

Thirdly, this research implemented the analysis of keywords of reasons for students’ respective opinions (Figure 4, Figure 5 and Figure 6). The research obtained the results of the word cloud and the frequency graph, with the top 20 keywords translated into English. As a result of the analysis, this research found that three opinions shared several keywords, namely “open book exam”, “study”, “thinking” and “problem” (Figure 4). In most opinions of agreement, different keywords were “opinion”, “use” and “knowledge”, while opinions of opposition showed keywords such as “concept” and “time”. In the last opinion, keywords were “courses”, “concepts”, “cases” and “contents”. This research utilized them as reference words in the fourth analysis.

This study analyzed the keywords from the reasons suggested by the students in their review opinion (excellence, something to improve) after the real time and online open book exam (Figure 5). As they wrote all their opinions together, this research analyzed keywords from all answers collectively rather than from each opinion. As a result of the analysis, the main keywords were “problem”, “thinking”, “difficulty”, “time”, “contents” and “number of problems”.

Then, this study analyzed the keywords from the reasons suggested by the students’ respective opinions of agreement, objection and another opinion toward a flexible pass grading system (Figure 6). As a result of the analysis, three opinions shared the main keywords of “thinking”, “opinion”, “student” and “grade”. In most opinions of agreement, the top keywords were “opinion”, “use”, “knowledge”, “cheating”, “situation” and “non-face-to-face”, while opinions of disagreement showed keywords such as “objection”, “study”, “exam” and “grading inflation”. In the last opinion, other keywords were “agreement”, “academic” and “the count of the grading system”.

Lastly, this research performed content analysis based on the keywords from the word cloud in three topics of the open book exam, real time and online exam and a flexible pass grading system. On average, students presented 1.43 (=156 comments/109 students) reasons for the open book exam, 2.42 reasons for the online open book exam and 1.39 reasons for the grading system (Figure 1).

**Figure 7:**
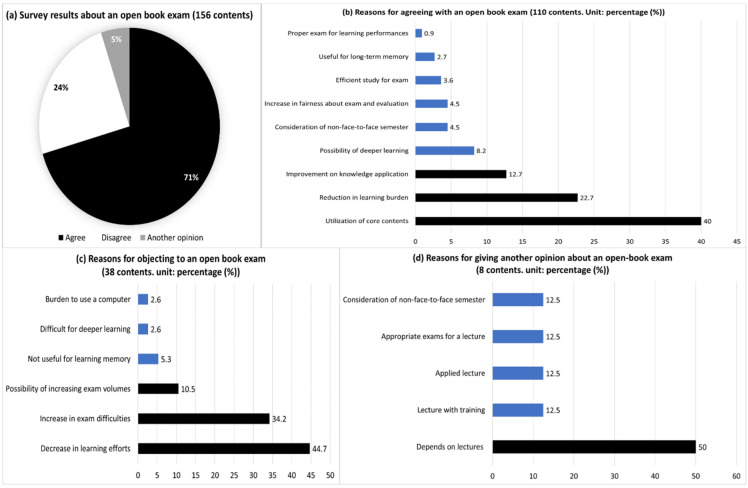
Opinions and answers about open book exams. Notes. Each figure’s totals may not equal 100 due to multiple answers and round-off decimals.

When analyzing students’ opinions of the open book exam and their reasons among 156 comments from 109 students, this research found that many related to agreement (71% in Figure 7). The main reasons for agreement (110 comments) were the utilization of core knowledge in the course content (40%), a reduction of the learning burden (22.7%), the improvement of knowledge application (12.7%), a possibility of deeper learning (8.2%) and the consideration of the difficulties in non-face-to-face learning (4.5%). The key reasons for opposition (38 comments) included a decrease in learning effort (44.7%), an increase in exam difficulty (34.2%) and an increase in exam/problem volume (10.5%). Other reasons for another opinion (8 comments) included that it was appropriate to adopt an open book exam according to the course content or difficulty level.

**Figure 8:**
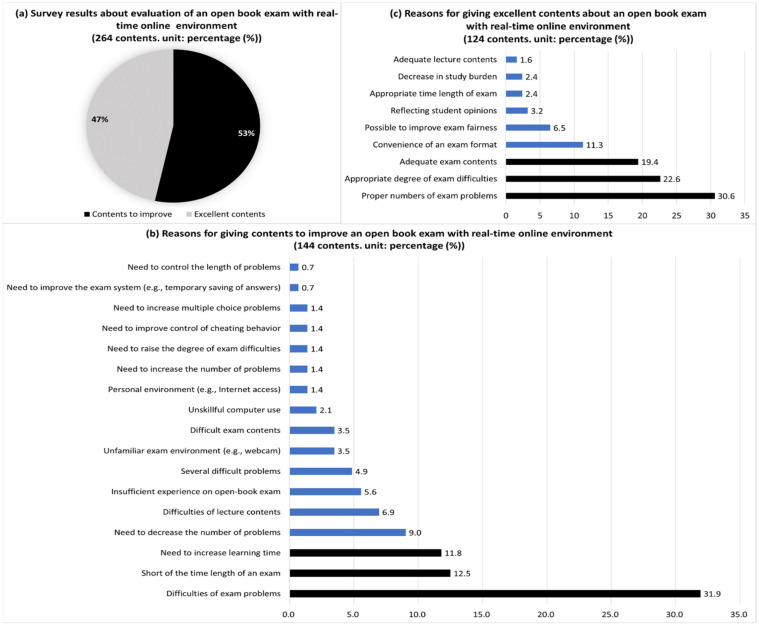
Opinions of excellence and/or something to improve about the exam after taking an open book final exam with a real time and online environment. Notes. Each figure’s totals may not equal 100 due to multiple answers and round-off decimals.

In the results of students’ opinions after taking the real time and online open book exam, this study found that many comments (53%) of the total 264 were related to improving the exam (Figure 8). The main reasons for improvement (144 comments) were students’ difficulty solving problems (31.9%), insufficient exam time (12.5%), feeling that they had to study more (11.8%), complaints about many problems (9%) and difficulty in non-face-to-face courses (6.9%). The key reasons for excellence (124 comments) were the appropriate number of problems (30.6%), proper levels of problem difficulties (22.6%), not-so-difficult content of questions (19.4%) and convenience of the exam format (e.g., Google-formatted survey, 11.3%).

**Figure 9:**
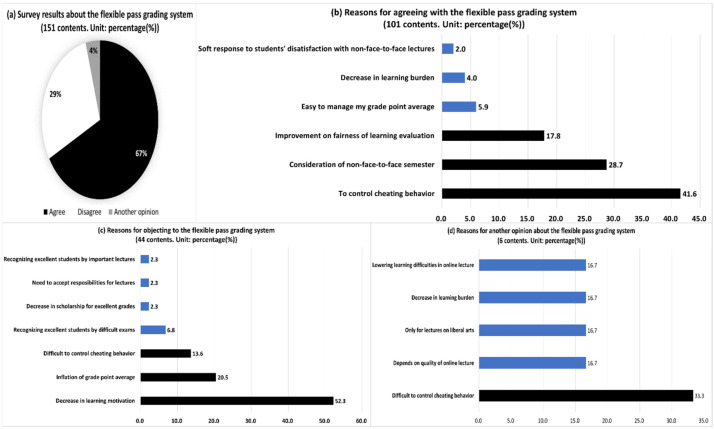
Opinions and answers about a flexible pass grading system. Notes. Each figure’s totals may not equal 100 due to multiple answers and round-off decimals.

This research finally found many comments related to agreement in the opinions of a flexible pass grading system and the reasons for these opinions (all 151 comments. Figure 9). The main reasons for agreement (101) were the control of cheating (41.6%), the consideration of difficulties in non-face-to-face learning (28.7%), the improvement of fairness in learning evaluation (17.8%) and the easy management of grade point average (5.9%). The key reasons for opposition (44 comments) were the decline in learning motivation or will (52.3%), inflation of grades (20.5%), the possibility to identify excellent students through difficult exams (6.8%) and the reduction in the opportunity to receive scholarship for excellent grades (2.3%). Other reasons for another opinion (6 comments) were difficulties in controlling fraudulent behavior (33.3%) and the conditional application of the grading system by course (e.g., nonmajor course, quality of non-face-to-face courses, 16.7%).

## 5. Discussion

### 5.1. Key Findings and Interpretations

Of the three research questions (i.e., RQ 1–3), students’ opinions of the open book exam and their reasons (RQ 1) were more likely to agree (70.6% out of 109). The main reasons for their agreement were the use of core knowledge from a course and the improvement of their application ability, the consideration of the situation of remote semesters and their learning burden and the promotion of fairness in learning evaluation. The key reasons for objection (22.9%) were the reduction in their learning time and effort compared to closed book exams, the increasing difficulties and length in the exam caused by more complex test questions and the burden of using computers.

These findings can suggest that many students support open books as they can lower their learning burden. The reason students approve of open book exams is related to the practical benefit of reducing learning burdens such as their stress and efforts to memorize course knowledge [18,19]. In addition, the exam focuses on assessing problem-solving capability using knowledge rather than memorization of the knowledge itself [19]. It can also help students gain experience in finding information about problems and obtaining creative answers in a realistic situation [18].

After taking the real time and online open book exam, students expressed more opinions favorable to improving the exam (53.4% of 264 comments. RQ 2). The main suggested improvements concerned their difficulties and lack of time, the need to increase the learning time for exam preparation (11.8%), the unfavorable environment of remote courses and unfamiliarity with the exam environment (e.g., the setting-up and the use of webcam, 3.5%). The key comments of excellence (46.6%) were the appropriate level of exam difficulty and the number of problems, the convenience of the exam format (e.g., Google-formatted survey, 11.3%), an instructor’s efforts to ensure fairness in learning evaluation (e.g., different problem sets (A, B, or C)), the use of webcam per student and the repetitive warning about cheating and grading policies (6.5%).

This finding can imply that students had insufficient preparation for the exam and limited experience with the test, despite their strong preference for the open book exam (70.6% in RQ 1). In an open book exam, students can face more difficulties than expected due to their unfamiliarity with the exam; they may spend too much time searching content of reference materials and experience tension in allocating time to writing answers during the exam [18]. In particular, students can acquire lower scores on online and remote exams than on face-to-face exams due to their lack of experience with the exam and various technical problems or concerns such as internet access, computer equipment instability and test software problems [32].

When administering real time and online open book exams, it is beneficial for instructors to provide students with ample relevant experience or information (e.g., pre-exams, exercises or sufficient tutorials) [32]. In these online courses, maintaining close interaction between instructors and learners is crucial for enhancing various aspects of learning recognition (e.g., teaching presence, cognitive presence, social presence) [33]. In some points, students could view themselves as consumers of courses rather than learners, as they can choose courses for their grades and future jobs and evaluate their satisfaction based on the ease of learning [34]. When preparing and implementing online open book exams, instructors need to persuade and change students’ thoughts on learning; the exam can enhance deeper learning of course knowledge by realizing learning opportunities of practicing, feedback and transparency in teaching and learning instead of forcing the memorization of course content. This process and interaction in terms of learning can develop students’ and instructors’ metacognitive thinking regarding learning and the exam.

Students’ positions (RQ 3) on a flexible pass grading system and their reasons showed that supporters (67%) outnumbered others. The main reasons for this favor were the possibility of a decrease in cheating behavior and the expectation of easier management of their learning and course grades. The key reasons for objection (29%) included a decline in their motivation for learning, the expectation about classmates’ increasing grade point averages and fierce competition for scholarship based on excellent grades and the doubt about the system’s effectiveness in controlling cheating behavior. Another opinion (4%) also highlighted the weak control of cheating behavior and the necessity for the limited use of the grading system.

### 5.2. Implications for Higher Education

Instructors and universities need to understand and guide their students, as young students or pupils with low grade point averages can be prone to attempt cheating in remote exams [16]. Because some students in other countries (e.g., South Korea, Cyprus, Australia and India) may not have sufficient experience with online learning and open book exams, they could exhibit a lack of self-control in multitasking (e.g., engaging in other activities while using social network services) or have low self-confidence in terms of exam preparation [1,2,12,35]. These issues of controlling cheating behavior and proctoring exams could be some of the most important factors for utilizing eExam (e.g., real time online open book tests) in many universities, even amid the educational challenges posed by COVID-19.

This research could be meaningful as the empirical analysis of the relevant policies about exams and the grading system, which are universities’ managerial policies about courses at a micro-level under the COVID-19 lockdown situation. This research also suggests that higher education institutions must develop some standard exam formats as well as various options for obtaining learning outcomes (e.g., grades) in the rapidly changing situation of decreasing students caused by low birth rates and by their higher expectations about university courses competing with private courses (e.g., MOOC, YouTube). For example, these changes in online learning can offer some developing countries (e.g., India, Nigeria) that have regional disparities in terms of internet access and educational devices (computers) valuable opportunities for educational innovation (e.g., transformation from offline to online materials, leading to cost saving) [12,36].

## 6. Conclusions

This research analyzed students’ perception and reasons regarding the open book exam and flexible pass grading system, which are ongoing policies of university education due to the recent COVID-19 pandemic. The study utilized several methodologies to obtain meaningful findings about students’ judgment and preference. The main findings revealed that many students supported the exam and the system as compensation for their learning difficulties caused by remote courses, control of cheating, and decline in course quality. Conversely, some students presented negative opinions, arguing that the exam and the system could not adequately control classmates’ cheating behavior and could paradoxically lead to a decrease in learning motivation. As another opinion, a few students conditionally supported the two systems, considering their unique circumstances of remote courses and learning environments. This research could be meaningful as one of empirical studies to investigate students’ recognition and related core concepts as educational consumers of the exam and grading system in a long-term and remote course environment.

This study has several limitations concerning sample size and methodologies (e.g., qualitative analysis with interviews) for the generalization of findings. By enhancing samples and methods, future research could explore appropriate exams and learning evaluation methods (e.g., structural equation modelling, sentiment analysis with student interviews) for students in the new normal of remote learning and our post-COVID environment. As the COVID-19 crisis appears to be relevant to the negative effects of excessive globalization and global warming, the topics of sustainable management considering energy and environment [37], management for the environment, social and corporate governance (ESG. [38,39]) would be promising and innovative areas for addressing our current learning challenges.

Furthermore, generative artificial intelligence (e.g., ChatGPT with GPT-4, Bard) and its various applications have dramatically changed students’ and instructors’ environments and behavior [40,41], which could require high educations to adopt innovative and interactive learning instead of relying solely on closed-book exam (e.g., multiple-choice tests) and/or short essays. In addition to the growing popularity of AI and online learning, it could be crucial for countries to ensure that their learners sufficiently understand local real-world issues (e.g., global trade and economic impact [42]) and strengthen learning contents relevant to their communities.

## Figures and Tables

**Table 1:** Descriptive statistics of demographic factors.

Panel A. Numeric Variables of Demographic Factors					
Variables	Observations	Mean	Standard Deviation	Minimum	Maximum
Scholastic years	109	3.385	1.845	1	8
Number taking an identical instructor’s courses	109	0.459	0.688	0	2
**Panel B. Categorical variables of demographic factors**					
**Variables**		**Observations**		**Percentage (%)**	
Major: Management, other including natural sciences and engineering		89, 20		81.7, 18.3	
Gender: female, male		52, 57		47.7, 52.3	
6 different courses		34, 8, 10, 15, 15, 27		31.2, 7.3, 9.2, 13.8, 13.8, 24.8	
Nationality: non-Korean, Korean		2, 107		1.8, 98.2	
Semester: spring, fall		52, 57		47.7, 52.3	

**Table 2:** Difference between demographic variables and agreement/disagreement with survey questions: nonparametric tests.

Panel A. Tests about Normal Distribution					
Tests	Observations	Agreement/Disagreement with an Open Book Exam		Agreement/Disagreement with a Flexible Pass Grading System	
		Test-Statistics	*p*-Value	Test-Statistics	*p*-Value
Jarque–Bera test	109	23.58 (chi-squared)	$0.000{\;}^{+++}$	18.24 (chi-squared)	$0.000{\;}^{+++}$
Shapiro–Francia W’ test	109	2.314 (z-score)	${0.010\;}^{++}$	−0.407 (z-score)	0.658
**Panel B. Equality tests about variables**					
**Opinion variables** **Demographic variables**	**Observations**	**Agreement/Disagreement with an Open book Exam**		**Agreement/Disagreement with an Open book Exam**	
		**Test-Statistics**	***p*-value**	**Test-Statistics**	***p*-value**
Semester 1 or 2 * W test	52, 57	−1.146 (z-score)	0.148	2.224 (z-score)	${0.026\;}^{++}$
Major: management or not. * W test	20, 89	−4.106 (z-score)	${0.000\;}^{+++}$	0.228 (z-score)	0.820
Gender: female or not. * W test	52, 57	0.609 (z-score)	0.543	0.36 (z-score)	0.719
Nationality: foreign or not * W test	2, 107	−0.907 (z-score)	0.365	−1.072 (z-score)	0.284
Different courses (i.e., 1–6) * KW test	34, 8, 10, 15, 15, 27	10.339 (chi-squared)	${0.066\;}^{+}$	5.034 (chi-squared)	0.412
		16.277 (chi-squared)	$0.006{\;}^{+++}$ (ties)	7.087 (chi-squared)	0.214
Scholastic years (i.e., 1–8) * KW test	16, 31, 13, 18, 15, 9, 5, 2	4.869 (chi-squared)	0.676	13.154 (chi-squared)	0.680
		7.666 (chi-squared)	0.363 (ties)	18.518 (chi-squared)	$0.010{\;}^{+}$
Number taking an identical professor’s courses (i.e., 1–3) * KW test	71, 26, 12	1.504 (chi-squared)	0.471	2.272 (chi-squared)	0.321
		2.368 (chi-squared)	0.306 (ties)	3.198 (chi-squared)	0.202

* Notes. W test: Wilcoxon rank-sum (or Mann–Whitney U) test. KW test: Kruskal–Wallis test. Significance level: 0.05. +++: *p*-value < 0.01, ++: *p*-value < 0.05, +: *p*-value < 0.1.

**Table 3:** Results of multinomial logistic regression: dependent variables of the open book exam and the pass grading system.

Independent Variables		Dependent Variable: Open Book Exam		Dependent Variable: Pass Grading System	
		Coefficient	Standard Error	Coefficient	Standard Error
**Opinion 1** **(Agreement)** * Base: another opinion	M1 (dummy)	0.441	1.032	−0.119	1.017
	Y18	−0.089	0.278	−0.182	0.526
	L2	−0.721	0.509	0.340	1.421
	S1 (dummy)	$1.698{\;}^{++}$	0.840	0.591	1.788
	F1 (dummy)	1.536	1.203	−0.361	1.511
	Fn1 (dummy)	$13.647{\;}^{+++}$	1.171	$13.790{\;}^{+++}$	0.988
	Constant	1.597	1.644	3.412	2.877
**Opinion 2** **(Objection)** * Base: another opinion	M1 (dummy)	$-2.054{\;}^{+}$	1.103	0.115	1.111
	Y18	−0.370	0.337	−0.004	0.531
	L2	−1.023	0.730	0.330	1.421
	S1 (dummy)	1.412	0.986	1.735	1.802
	F1 (dummy)	1.393	1.315	0.171	1.535
	Fn1 (dummy)	−0.276	1.035	−0.047	0.955
	Constant	$3.572^{+}$	1.911	1.087	2.937
**Model statistics**	Observation	109	-	109	-
	Wald chi-square	$403.80{\;}^{+++}$	Degree of freedom: 12	$336.47^{+++}\;$	Degree of freedom: 12

* Notes. Open (opinions about an open book exam), pass (opinions about a flexible pass grading system), M1 (major: management = 1), Y18 (scholastic years: 1–8), L2 (attending different courses: 0–2), F1 (female = 1), Fn1 (foreign student = 1), S1 (fall semester = 1). Significance level: 0.05. +++: *p*-value < 0.01, ++: *p*-value < 0.05, +: *p*-value < 0.1. This research omitted the result about the marginal effects of the multinomial logistic regression due to length limitation.

## Data Availability

The data presented in this study are available on request from the corresponding author.
